# Comparative Genomics Analysis Reveals the Genomic Basis of S8 Proteases, CAZymes, and Secondary Metabolism Associated with Nematode Biocontrol in *Purpureocillium lilacinum*

**DOI:** 10.3390/ijms27114687

**Published:** 2026-05-22

**Authors:** Xiaoxi Cheng, Li Liu, Zhimin Zhu, Minghao Chen, Wenbo Wang, Jialin Li, Ramon Santos Bermudez, Xiujun Zhang, Wenxing He

**Affiliations:** School of Biological Science and Technology, University of Jinan, Jinan 250000, China

**Keywords:** *P. lilacinum*, comparative genomics, plant-parasitic nematodes, CAZymes, S8 serine protease, secondary metabolite gene clusters

## Abstract

Biological control fungi play an important role in the management of plant-parasitic nematodes; however, the molecular basis underlying their diverse biocontrol strategies remains incompletely understood. In this study, a comparative genomic analysis was performed on four representative biocontrol fungi: *Purpureocillium lilacinum* PLFJ-1, *Trichoderma harzianum* CBS 226.95, *Pochonia chlamydosporia* 170, and *Aspergillus niger* CBS 513.88. Genome comparison revealed substantial variation: genome size ranged from 34.0 Mb (*A. niger*) to 44.2 Mb (*P. chlamydosporia*), GC content from 47.5% (*T. harzianum*) to 58.5% (*P. lilacinum*), and predicted gene models also differed markedly among the four fungi. Phylogenetic analysis based on the Internal Transcribed Spacer divided these fungi into two major clades corresponding to distinct evolutionary lineages. Orthogroup analysis identified both a conserved core gene set and species-specific gene repertoires. Functional annotation using KEGG, KOG, and GO indicated a high degree of conservation across core metabolic processes, catalytic activities, and cellular components, with distinct differences within specific functional categories. Further comparative analyses demonstrated pronounced variation in the composition and abundance of carbohydrate-active enzymes (CAZymes) and peptidases, as well as a notable expansion and enrichment of S8 subtilisin-like serine peptidases in the nematode-parasitic fungi *P. lilacinum* and *P. chlamydosporia*. Secondary metabolite analysis revealed lineage-specific biosynthetic gene clusters (BGCs). Notably, *P. lilacinum* and *P. chlamydosporia* carried PKS/NRPS clusters potentially linked to nematicidal activity, while *A. niger* and *T. harzianum* displayed broader but less infection-specific metabolic profiles. Together, these findings suggest that distinct enzymatic and metabolic gene repertoires, particularly expansions of S8 serine peptidases and specific CAZyme families, may contribute to the biocontrol potential of these fungi.

## 1. Introduction

Over recent decades, rapid global population growth has placed increasing pressure on agricultural production, creating an urgent need for efficient and sustainable crop protection strategies. Root-knot nematodes (RKNs) are major plant-parasitic nematodes worldwide, infecting a wide range of crops, including staple foods, vegetables, and medicinal plants, and causing substantial economic losses. It is estimated that four major species-*Meloidogyne incognita*, *Meloidogyne arenaria*, *Meloidogyne javanica*, and *Meloidogyne hapla*-collectively account for approximately US$100 billion in annual global agricultural yield losses, with economic damage exceeding US$1 billion per year in the United States [1]. In many agricultural systems, RKN infestation causes substantial yield losses and quality deterioration across diverse crops, with infection rates exceeding 80% and yield reductions of 50–80% in severe cases [2,3,4].

Current control strategies for RKNs remain limited. Agronomic practices provide only partial suppression due to the broad host range and environmental adaptability of these nematodes. Meanwhile, chemical nematicides raise concerns regarding environmental contamination, human health risks, and resistance development. Physical control methods, such as soil solarization [5], are environmentally friendly but require prolonged treatment and are difficult to implement at large scales [5,6,7,8,9,10]. Given these limitations, biological control has emerged as a viable alternative [11]. Nematophagous fungi, as natural enemies of nematodes, suppress nematode populations through secreting extracellular enzymes and trapping or parasitic mechanisms, and can be classified into four groups: (i) predatory fungi, (ii) endoparasitic fungi, (iii) egg-parasitic fungi, and (iv) toxin-producing fungi [11,12,13,14].

Among them, *P. lilacinum* (formerly known as *Paecilomyces lilacinus*) is one of the most widely studied and commercially applied biocontrol fungi. Previous research has demonstrated that *P. lilacinum* infects the eggs and adult females of root-knot nematodes, secretes extracellular hydrolytic enzymes, such as proteases and chitinases, and produces secondary metabolites that enhance nematode mortality [15,16,17,18,19].

However, to date, the molecular mechanisms underlying the pathogenicity of *P. lilacinum* toward nematodes remain poorly elucidated. Previous studies have demonstrated the involvement of extracellular enzymes, including proteases, collagenases, and chitinases, as well as secondary metabolites/bioactive compounds, which are essential for their biocontrol attributes [20,21]. However, systematic molecular characterization remains limited, with only a small number of individual genes, including one serine protease [20] and one keratinase [22], having been functionally characterized to date.

However, systematic comparative genomic analyses among these species remain limited, particularly for key functional modules such as carbohydrate-active enzymes, serine proteases, and secondary metabolite biosynthetic gene clusters.

In this study, we performed a comparative genomic analysis of *P. lilacinum* PLFJ-1, *T. harzianum* CBS 226.95, *P. chlamydosporia* 170, and *A. niger* CBS 513.88. *P. lilacinum* and *P. chlamydosporia* are well-known nematode-parasitic fungi. Meanwhile, *T. harzianum* is a widely studied biocontrol agent with antagonistic activity. *A. niger* was included as a non-biocontrol saprophytic reference species. By integrating genome assembly, phylogenetic analysis, orthogroup comparison, functional annotation, and detailed investigation of CAZymes, peptidases, and secondary metabolite gene clusters, we aimed to characterize both conserved and species-specific genomic features associated with biocontrol potential. This study provides a systematic framework for understanding the molecular basis of nematode parasitism and a genomic foundation for the rational improvement and application of highly efficient *P. lilacinum*-based biocontrol agents.

## 2. Results

### 2.1. Assembled Genomic Features and General Comparison

To compare the general genomic features of the four fungi, genome assembly statistics and annotation data were collected and analyzed under a unified framework. Comparative analysis of the genome assembly revealed substantial variation among four biocontrol fungi in genome size, GC content, and assembly contiguity (Table 1). Genome sizes ranged from 34 Mb in *A. niger* CBS 513.88 to 44.2 Mb in *P. chlamydosporia* 170. *A. niger* CBS 513.88 possessed the smallest genome (34 Mb), consisting of 19 scaffolds with a scaffold N50 of 2.5 Mb. In contrast, *P. lilacinum* PLFJ-1 exhibited a larger genome (38.5 Mb) but a more fragmented assembly (163 scaffolds), despite a comparable scaffold N50 (3.2 Mb). Notably, *T. harzianum* CBS 226.95 possessed a larger genome (41 Mb) with the highest number of scaffolds (532), suggesting lower assembly contiguity despite a relatively high contig N50 (360.6 kb). By contrast, *P. chlamydosporia* 170 possesses the largest genome (44.2 Mb) and exhibits the highest assembly quality. It contained only 49 scaffolds, with a scaffold N50 of 5.4 Mb and an L50 of 2, indicating the highest assembly contiguity among the four genomes.

GC content varied considerably, ranging from 47.5% in *T. harzianum* to 58.5% in *P. lilacinum*, suggesting lineage-specific genomic features and evolutionary adaptations. Overall, these variations in genome size and assembly quality may impact the complexity of gene repertoires, particularly for gene families linked to parasitism and environmental adaptation.

### 2.2. Phylogenetic Framework and Species Selection

**Figure 1:**
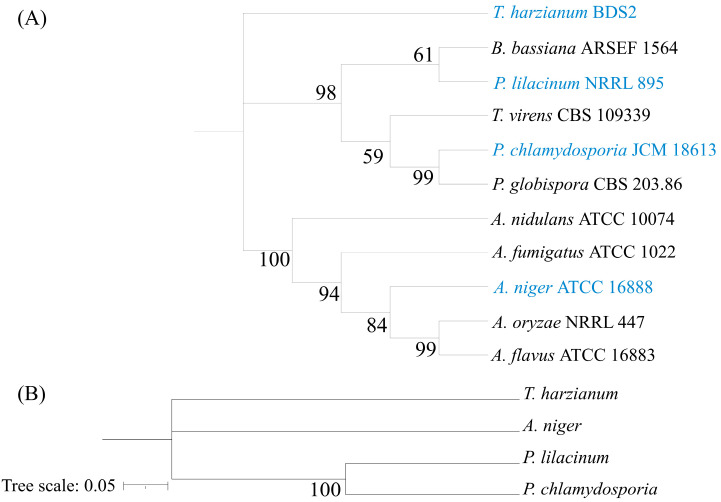
(**A**) ITS-based phylogenetic tree of 11 fungal species used for preliminary phylogenetic framework construction and representative species selection. Bootstrap values are shown at branch nodes as percentages of 1000 replicates. Only bootstrap values greater than 50% are indicated. The scale bar represents 0.05 substitutions per site. (**B**) Maximum-likelihood phylogenomic tree constructed using concatenated alignments of 200 conserved single-copy orthologous proteins from the four selected fungal genomes. Bootstrap support values are indicated at the nodes.Species highlighted in blue indicate the four fungal genomes selected for subsequent comparative genomic analyses.

To establish a broad phylogenetic framework for species selection in comparative genomic analyses, phylogenetic analysis of 11 fungal species was initially performed based on Internal Transcribed Spacer (ITS) sequences (Figure 1A). The selected taxa represented diverse ecological lifestyles, including nematophagous, entomopathogenic, and biocontrol-associated fungi, as well as saprophytic *Aspergillus* species. The resulting tree provided a preliminary overview of phylogenetic relationships among these fungi. It supported the selection of four representative species, namely *P. lilacinum*, *P. chlamydosporia*, *T. harzianum*, and *A. niger*, for subsequent comparative genomic analyses.

To obtain a more robust phylogenomic framework for comparative analyses, a maximum-likelihood phylogenetic tree was constructed from concatenated alignments of 200 conserved single-copy orthologous proteins identified in the four selected fungal genomes (Figure 1B). The phylogenomic tree showed that *P. lilacinum* and *P. chlamydosporia* formed a closely related clade, consistent with their shared nematophagous lifestyle. In contrast, *A. niger* occupied a more distant evolutionary position, while *T. harzianum* formed a separate lineage among the analyzed fungi.

These phylogenomic relationships may partially reflect ecological adaptation and similarities in host-associated lifestyles among the selected fungi, and provide a robust evolutionary framework for subsequent comparative analyses of gene families associated with parasitism, host interaction, and biocontrol potential, including CAZymes, peptidases, and secondary metabolite-related genes.

### 2.3. Comparative Analysis of Orthogroups

**Figure 2:**
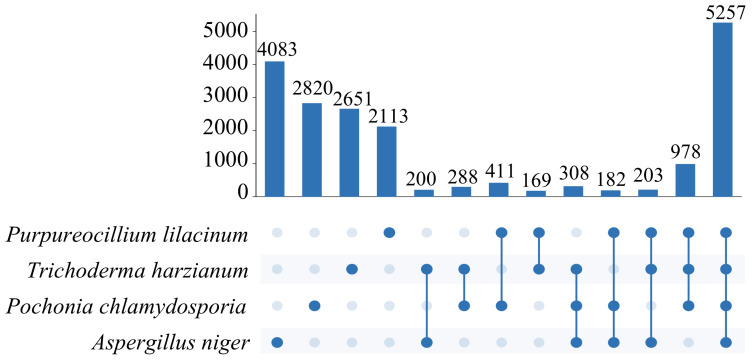
UpSet plot showing the shared and unique orthogroups among *P. lilacinum* PLFJ-1, *T. harzianum* CBS 226.95, *P. chlamydosporia* 170, and *A. niger* CBS 513.88. The vertical bars represent the number of orthogroups in each intersection, while the horizontal bars indicate the total orthogroups in each species.

To identify conserved and species-specific gene families, orthologous gene analysis was performed using OrthoFinder based on protein sequences from the four genomes. Comparative analysis of orthologous gene families among the four fungal species identified a total of 19,838 orthogroups. Detailed category counts are provided in Supplementary Table S1. Of these, 5257 orthogroups were shared across all four genomes, representing the conserved genetic core of these fungi (Figure 2). Among this shared core, numerous orthogroups were distributed among specific subsets of species. Notably, the three biocontrol-associated fungi (*P. lilacinum*, *T. harzianum*, and *P. chlamydosporia*) shared more orthogroups with each other than with *A. niger*, suggesting a closer functional and evolutionary relationship among these antagonistic or parasitic species. This distinction provides a comparative framework to separate biocontrol-related genomic features from those associated with general saprophytic lifestyles, thereby improving the biological interpretability of subsequent analyses. Species-specific orthogroups were also identified in each genome. Specifically, 4082, 2113, 2651, and 2820 unique orthogroups were identified in *A. niger*, *P. lilacinum*, *T. harzianum*, and *P. chlamydosporia*, respectively (Figure 2). These unique gene sets may contribute to ecological specialization, particularly traits associated with host interaction, environmental adaptation, and niche differentiation. In summary, UpSet analysis identified a conserved genomic foundation alongside variable, lineage-specific gene sets, highlighting significant differences in gene family composition among the four fungal species.

### 2.4. Functional Annotation and Classification

**Figure 3:**
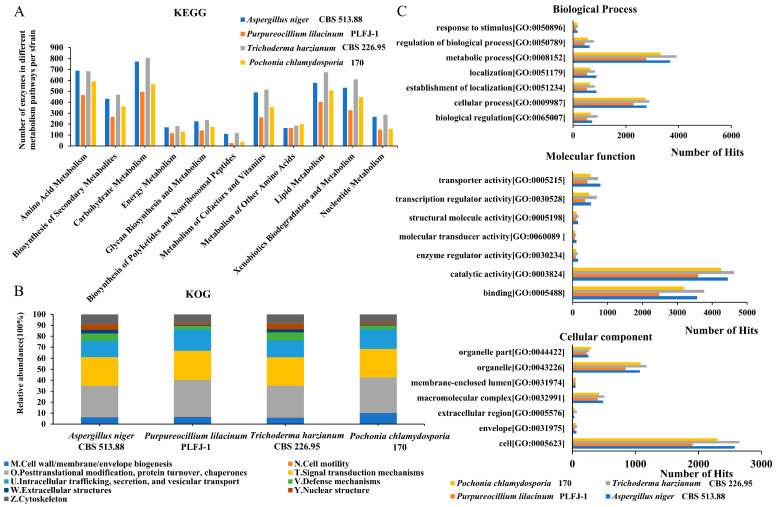
Functional classification of predicted proteins based on KEGG, KOG, and GO annotation. (**A**) KEGG pathway categories. (**B**) KOG functional classification. (**C**) GO annotation across biological process, molecular function, and cellular component categories.

To characterize the functional composition of the four fungal genomes, protein sequences were annotated using KEGG, KOG, and GO databases, and the distribution of functional categories was compared across species. Comparative analysis based on Kyoto Encyclopedia of Genes and Genomes (KEGG) pathway annotations revealed pronounced differences in metabolic functional composition among the four fungal genomes (Figure 3A). Despite these differences, amino acid metabolism and carbohydrate metabolism were the most represented functional categories across all four fungi, indicating a high degree of conservation in core nutritional metabolic processes. Among the four species, *A*. *niger* and *T. harzianum* exhibited relatively higher numbers of annotated enzymes across most metabolic pathways, particularly in secondary metabolite biosynthesis, lipid metabolism, and xenobiotic biodegradation and metabolism. This pattern reflects their more complex metabolic networks and enhanced capacity for environmental adaptation. In contrast, *P. lilacinum* and *P. chlamydosporia* displayed relatively lower overall numbers of annotated metabolic pathways, although genes involved in glycan biosynthesis and amino acid metabolism remained well represented.

Comparative analysis of genome functional composition based on Eukaryotic Orthologous Groups (KOG) classification revealed a highly similar overall distribution pattern across major functional categories, with variation in the relative abundance of specific functional groups (Figure 3B). In terms of relative abundance of specific functional categories, the category “posttranslational modification, protein turnover, and chaperones” represented a substantial proportion in all species. “Signal transduction mechanisms” and “intracellular trafficking, secretion, and vesicular transport” were also consistently abundant across the four genomes. Differences among species were observed in several categories. The relative proportions of “cell wall/membrane/envelope biogenesis” varied among the strains, with *P. chlamydosporia* and *P. lilacinum* exhibiting higher representation in this category. In contrast, the category “defense mechanisms” showed relatively stable representation in *A. niger* and *T. harzianum.* In contrast, the categories “cell motility”, ”extracellular structures”, and “nuclear structure” were present at low proportions in all four species.

Gene Ontology (GO) functional annotation revealed differences in gene distribution among the four fungi across the three main categories: biological process, molecular function, and cellular component (Figure 3C). Within the “Biological Process” category, all four genomes were predominantly enriched in “metabolic process” (GO:0008152) and “cellular process” (GO:0009987). In addition, “regulation of biological process” (GO:0050789), “biological regulation” (GO:0065007), as well as “localization” (GO:0051179) and “establishment of localization” (GO:0051234) were also well represented. At the same time, “response to stimulus” (GO:0050896) was relatively less abundant with variation among species. Within the “Molecular Function” category, genes were predominantly enriched in “catalytic activity” (GO:0003824) and “binding” (GO:0005488), with smaller proportions assigned to “transcription regulator activity” (GO:0030528), “molecular transducer activity” (GO:0060089), and “transporter activity” (GO:0005215). Within the “Cellular Component” category, most genes were annotated to “cell” (GO:0005623) and “organelle” (GO:0043226), followed by “organelle part” (GO:0044422) and “macromolecular complex” (GO:0032991). By contrast, genes associated with the “extracellular region” (GO:0005576) and “membrane-enclosed lumen” (GO:0031974) were less abundant across all species.

**Figure 4:**
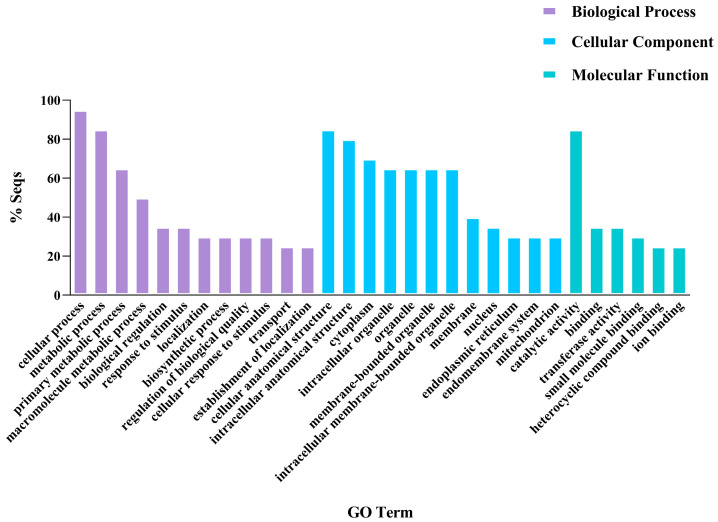
Gene Ontology (GO) enrichment analysis of species-specific genes in the four representative fungi. Enriched GO terms are shown for biological process (BP), molecular function (MF), and cellular component (CC) categories. The top significantly enriched terms (*p* < 0.05) are displayed.

To provide a more mechanistic understanding, functional enrichment analyses were further performed specifically on *P. lilacinum* species-specific genes. GO enrichment (Figure 4) revealed statistically significant overrepresentation of genes associated with proteolysis, serine-type peptidase activity, carbohydrate metabolic process, extracellular region, and transmembrane transport (*p* < 0.05). Detailed category counts are provided in Supplementary Table S2. These enriched functions are potentially associated with extracellular degradation, nutrient acquisition, and host interaction during nematode parasitism. Hydrolytic and proteolytic activities may facilitate nematode egg penetration and cuticle degradation, while enriched secondary metabolite pathways likely contribute to antimicrobial activity and ecological adaptation. Signal transduction and intracellular trafficking genes may play roles in host recognition and response. Together, these analyses provide a robust functional framework linking genomic features to ecological and biocontrol traits.

Overall, the four biocontrol fungi exhibited a high degree of similarity at the level of GO, KOG, and KEGG classification, with differences primarily reflected in the relative abundance of genes within specific functional categories, now further supported by statistical enrichment analyses and mechanistic interpretation.

### 2.5. CAZymes, Peptidases, and Phylogenetic Analysis of Key Hydrolytic Enzymes

**Figure 5:**
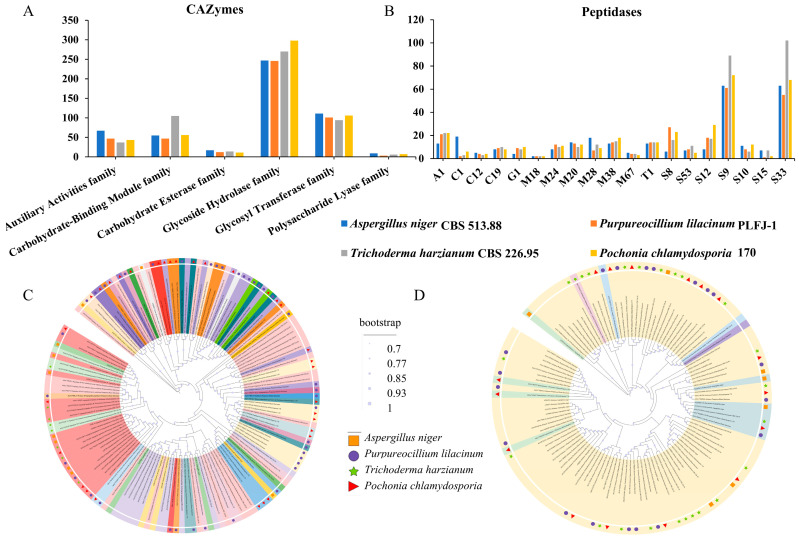
Comparative analysis of CAZymes and peptidases. (**A**) Bar plot showing the distribution of major CAZyme families. (**B**) Bar plot showing the distribution of peptidase families. (**C**) Phylogenetic tree of S8 serine proteases. (**D**) Phylogenetic tree of chitinases from different fungal species. Phylogenetic trees were constructed using the maximum likelihood method with 1000 bootstrap replicates; bootstrap values > 50% are shown at nodes.

To investigate enzymatic systems potentially involved in host interaction and biocontrol, CAZymes and peptidases were identified and classified based on the dbCAN2 and MEROPS databases, respectively (Figure 5A). Detailed category counts are provided in Supplementary Table S2. These enzymes were mainly assigned to the glycoside hydrolase (GH), glycosyltransferase (GT), carbohydrate esterase (CE), polysaccharide lyase (PL), carbohydrate-binding module (CBM) and auxiliary activity (AA) family. A total of 2054 CAZyme genes were identified. *T. harzianum* exhibited relatively higher numbers of GH and CBM family members, whereas *P. lilacinum* showed relative enrichment in GT and GH families.

Peptidases were systematically identified and classified based on the MEROPS database (Figure 5B). Detailed category counts are provided in Supplementary Table S3. The results showed that all four genomes encode multiple peptidase-related genes, including serine peptidases, metallopeptidases, cysteine peptidases, and aspartic peptidases. Among all analyzed strains, serine peptidases and metallopeptidases constituted the predominant peptidase types. Notably, *P. lilacinum* and *P. chlamydosporia* harbored relatively higher numbers of genes in the S8 and S9 serine peptidase families. Specifically, *P. lilacinum* and *P. chlamydosporia* encoded 27 and 23 S8 serine peptidases, respectively, representing an apparent expansion compared with *T. harzianum* and *A. niger*. In contrast, *T. harzianum* and *A. niger* exhibited higher gene abundances within metallopeptidase families. In addition, cysteine peptidases and aspartic peptidases were present in all four genomes. However, their overall numbers were relatively low, and the interstrain differences were less pronounced than those observed for serine peptidases and metallopeptidases.

To investigate the evolutionary relationships of key hydrolytic enzymes, phylogenetic analyses of S8 serine peptidases and chitinases were conducted based on their amino acid sequences using the Maximum Likelihood method implemented in IQ-TREE (Figure 5C,D; See Supplementary Material Figures S1 and S2 for a clearer version). The phylogenetic analysis of S8 serine peptidases showed that these proteins were grouped into several well-supported clades (Figure 5C). Members from *P. lilacinum* and *P. chlamydosporia* tended to cluster within certain clades and formed species-specific monophyletic subgroups, indicative of lineage-specific gene expansion. In contrast, S8 peptidases from *T. harzianum* and *A. niger* were distributed across multiple clades and frequently clustered with homologs from other species, indicating a more conserved evolutionary pattern. Phylogenetic analysis of chitinases revealed a different evolutionary trend (Figure 5D). Most chitinase proteins formed several conserved clades, with sequences from different fungal species interspersed, reflecting a relatively high degree of evolutionary conservation. Nevertheless, some clades contained a higher proportion of chitinase members from *P. lilacinum* and *P. chlamydosporia*, suggesting moderate species-specific aggregation and limited lineage-specific expansion.

### 2.6. Nutrient-Responsive Expression Patterns of Highly Secreted S8 Serine Proteases

Given the expansion and phylogenetic diversification of S8 serine proteases identified in *P. lilacinum*, four highly secreted S8 proteases were further selected for expression profiling under different nutritional conditions. This analysis was performed to investigate whether these proteases exhibit differential transcriptional responses to environmental carbon and nitrogen sources, potentially reflecting functional specialization associated with nutrient utilization and environmental adaptation.

**Figure 6:**
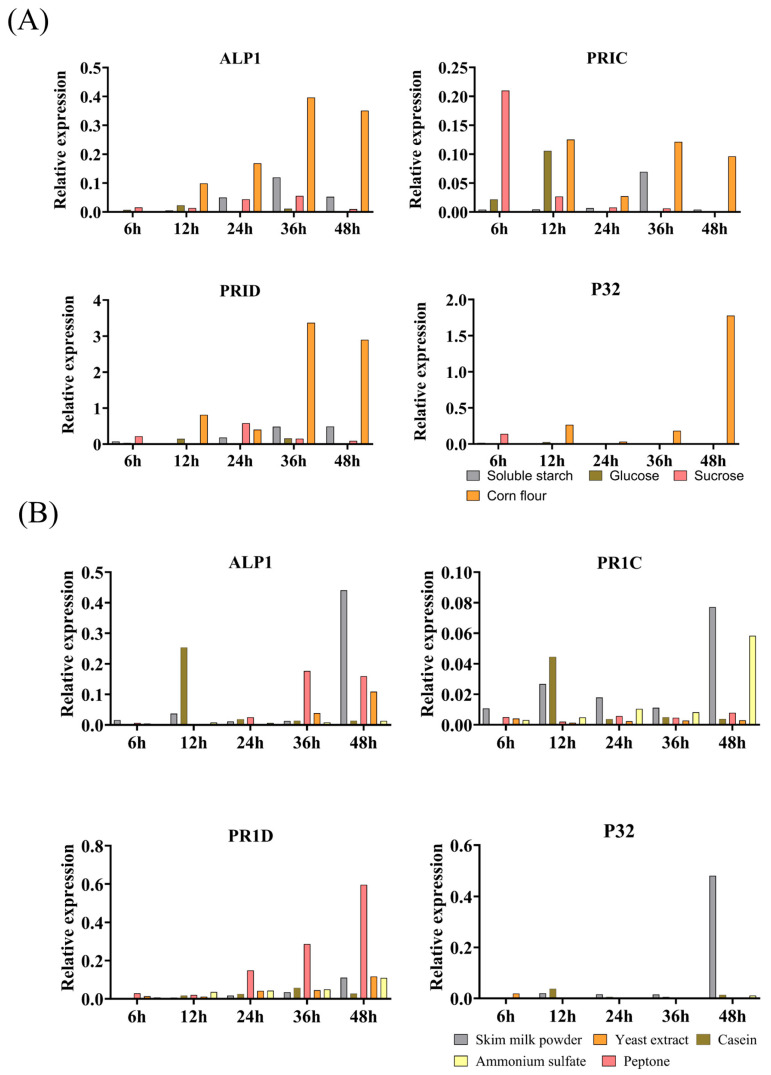
Expression levels of four highly secreted S8 serine protease genes (ALP1, PR1C, PR1D, P32) under different carbon (**A**) and nitrogen sources (**B**). Relative expression was quantified by qRT-PCR(Quantitative Reverse Transcription Polymerase Chain Reaction) and normalized to the reference *act* gene. Data represent mean ± SD of three biological replicates.

Expression analysis of four highly secreted S8 serine protease genes (Alp1, PR1C, PR1D, P32) [26] was performed under four different carbon sources: corn flour, soluble starch, sucrose, and glucose (Figure 6A). The Alp1 gene showed clear induction in the presence of corn flour, starting at 12 h and gradually increasing to a peak at 36–48 h (relative expression ≈ 0.40). In contrast, expression levels under glucose and sucrose remained very low throughout the fermentation period. PR1D exhibited the strongest induction, reaching a relative expression of 3.37 at 36 h under corn flour, significantly higher than the other genes. PR1C showed a transient peak at 6 h under sucrose, followed by a rapid decrease. P32 displayed delayed induction, with low expression before 36 h and a pronounced increase at 48 h under corn flour (relative expression 1.78).

Expression patterns under different nitrogen sources-skim milk powder, casein, peptone, yeast extract, and ammonium sulfate-were also evaluated (Figure 6B). Skim milk powder induced the broadest and strongest expression, particularly at 48 h, with Alp1 and P32 reaching relative expression levels of 0.44 and 0.48, respectively. PR1D expression was highest under peptone, showing a steady increase from 24 h to 48 h (peak 0.60). PR1C exhibited an early transient peak at 12 h under casein, followed by a rapid decline. Under ammonium sulfate, expression levels of Alp1, PR1D, and P32 remained near zero, consistent with nitrogen catabolite repression (NCR), whereas PR1C showed minimal induction at 48 h.

These results indicate that the four highly secreted S8 serine proteases exhibit differential transcriptional responses to carbon and nitrogen sources, suggesting potential functional specialization in substrate degradation and nutrient adaptation. Such nutrient-responsive regulation may also support their roles in host-associated interactions in nematophagous fungi.

### 2.7. Secondary Metabolite Biosynthetic Gene Clusters

**Figure 7:**
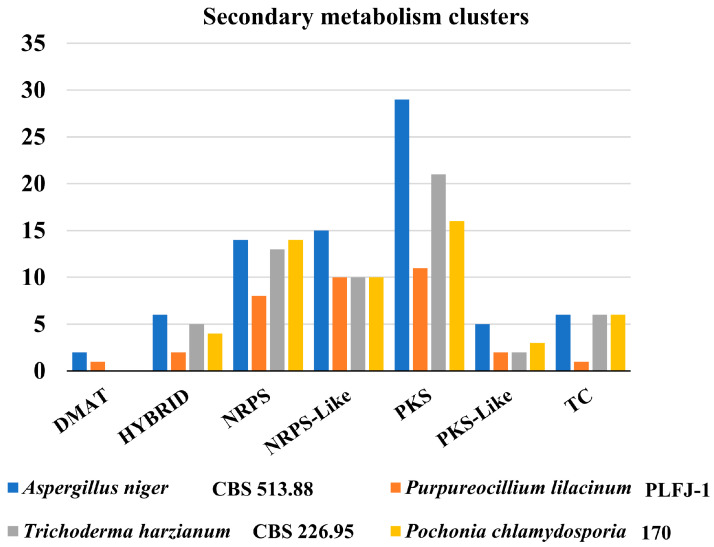
Distribution of secondary metabolite biosynthetic gene clusters in different fungal strains. Numbers of predicted gene clusters, including dimethylallyl tryptophan synthase (DMAT), hybrid, nonribosomal peptide synthetase (NRPS), NRPS-like, polyketide synthase (PKS), PKS-like, and terpene cyclase (TC) clusters, are shown for *A. niger* CBS 513.88, *P. lilacinum* PLFJ-1, *T. harzianum* CBS 226.95, and *P. chlamydosporia* 170.

Secondary metabolite biosynthetic gene clusters (BGCs) in the genomes of the four fungal species were systematically analyzed based on the predictions generated by the antiSMASH platform (Figure 7). Detailed category counts are provided in Supplementary Table S4. The results showed that all four genomes encoded multiple types of secondary metabolite biosynthetic gene clusters, mainly including polyketide synthase (PKS), nonribosomal peptide synthetase (NRPS), and PKS–NRPS hybrid clusters.

Comparative analysis revealed clear differences in both the number and composition of BGCs among the four fungi. A total of 77 (*A. niger*), 35 (*P. lilacinum*), 57 (*T. harzianum*), and 43 (*P. chlamydosporia*) BGCs were identified. Among the four genomes, clear variation was observed in the abundance of NRPS-like and PKS-like gene clusters, with *A. niger* exhibiting the highest numbers in both categories. In contrast, the secondary metabolite gene cluster profiles of the four fungi exhibited distinct distribution patterns, with PKS clusters representing the dominant category in several species. In addition to these major classes, other types of BGCs, including terpene and other hybrid clusters, were also identified across the four genomes, although their abundance varied among species. Overall, the diversity and composition of secondary metabolite gene clusters observed in the four fungal genomes may reflect differences in their metabolic capabilities and ecological adaptation strategies.

**Figure 8:**
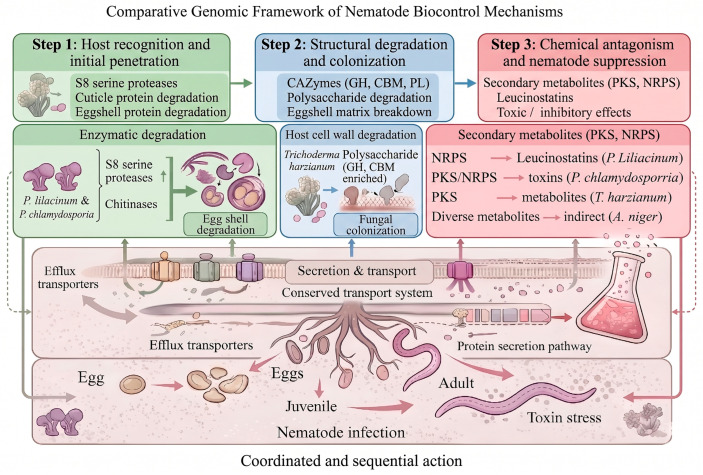
Hypothetical model illustrating the sequential and coordinated roles of S8 proteases, CAZymes, and secondary metabolism in nematode infection. Step 1: S8 serine proteases are involved in the degradation of proteinaceous structures such as the nematode cuticle and eggshell, facilitating initial host penetration. Step 2: Carbohydrate-active enzymes (CAZymes), including glycoside hydrolases and carbohydrate-binding modules, contribute to the breakdown of polysaccharide components in the eggshell and surrounding matrices, promoting fungal colonization. Step 3: Secondary metabolites, including polyketides and nonribosomal peptides (e.g., leucinostatins), may exert toxic or inhibitory effects on nematodes, leading to suppression or mortality.

These findings highlight the distinct metabolic potentials and secondary metabolite profiles that may contribute to the species-specific biocontrol characteristics of the four fungi. Figure 8 illustrates a stepwise mechanistic model derived from comparative genomic analyses. Specifically, S8 proteases may function in the initial stage of host penetration, followed by CAZymes that contribute to structural degradation and fungal colonization, while secondary metabolites may act in later stages by exerting nematicidal or inhibitory effects. This sequential and coordinated framework provides a plausible genomic basis for nematode biocontrol. This model illustrates the key functional modules associated with nematode antagonism, including the expansion and enrichment of S8 serine peptidases, differential composition of CAZyme families involved in cell wall and eggshell degradation, and species-specific distribution of secondary metabolite biosynthetic gene clusters. The potential synergy between hydrolytic enzymes and bioactive secondary metabolites may facilitate fungal penetration, host degradation, and nematicidal activity.

## 3. Discussion

Comparative genomic analysis revealed substantial variation in genome architecture and functional gene composition among the four fungi, reflecting their distinct evolutionary trajectories and ecological strategies. Consistent with this framework, our comparative analyses explicitly distinguish biocontrol-associated fungi from a saprophytic reference species, allowing the identification of gene families potentially linked to nematode parasitism. The larger genome and higher assembly completeness observed in *P. chlamydosporia* are consistent with previous genomic studies showing that parasitic fungi tend to possess expanded gene repertoires supporting host recognition, penetration, and nutrient acquisition [27,28,29,30]. In contrast, *A. niger* possesses a relatively compact genome but retains extensive metabolic capacity, a feature widely recognized as characteristic of saprophytic fungi with strong environmental adaptability [23,31]. *P. lilacinum* and *T. harzianum* displayed intermediate genome sizes but differed in GC content and functional gene composition, suggesting that distinct selective pressures have shaped their genomic architectures toward different ecological and biocontrol strategies [32,33].

Phylogenetic analysis further demonstrated that nematode antagonism is not restricted to a single fungal clade, but has evolved independently across multiple fungal groups [27,34]. The close phylogenetic relationship between *P. chlamydosporia* and *P. lilacinum* is consistent with earlier reports suggesting partial conservation of parasitism-related mechanisms within Hypocreales [32,35], whereas *T. harzianum*, despite its phylogenetic distance, highlights the importance of functional gene content rather than phylogenetic proximity alone [36]. Similar patterns of convergent evolution have been documented in other nematode-trapping fungi, such as *Arthrobotrys oligospora*, where distinct trapping mechanisms evolved independently across lineages [27,35].

In this study, mechanistic insight is inferred at the genomic level through the integration of functionally relevant gene families, rather than direct experimental validation. Functional annotation indicated that core metabolic pathways are conserved across the four fungi, whereas pathways associated with secondary metabolism and environmental interaction exhibit substantial variation [28,29]. The extensive metabolic networks observed in *A. niger* and *T. harzianum* are consistent with previous studies reporting that metabolic versatility enhances competitive fitness in heterogeneous soil and rhizosphere environments [23,30,37]. Conversely, *P. lilacinum* and *P. chlamydosporia* exhibited relatively streamlined metabolic profiles, which may reflect an evolutionary shift toward functional specialization associated with parasitic or host-adapted lifestyles [32,38,39]. Such metabolic specialization has been proposed as a strategy to allocate energetic resources preferentially toward infection-related processes rather than broad substrate utilization [38].

Secondary metabolite biosynthetic gene clusters (BGCs) further contribute to the functional differentiation among these fungi. Secondary metabolites, including polyketides and nonribosomal peptides, have been demonstrated to exhibit nematicidal or inhibitory activity against nematodes [28,32]. For example, *P. lilacinum* is known to synthesize leucinostatins, a class of peptide antibiotics with demonstrated biological activity against multiple pathogens, highlighting the potential role in nematode suppression [32,40]. The observed variation in the number and composition of BGCs among the four fungi suggests differences in their chemical arsenals and potential modes of antagonism. In particular, the presence of lineage-specific BGCs may indicate the evolution of specialized metabolites involved in host interaction.

One of the most prominent features observed in this study is the expansion of peptidases, particularly S8 serine proteases, in *P. lilacinum* and *P. chlamydosporia*. This pattern is consistent with previous studies demonstrating the role of subtilisin-like peptidases in fungal parasitism of nematodes [37]. In contrast, *T. harzianum* and *A. niger* harbored more genes belonging to metallopeptidase families, which are associated with extracellular protein degradation, nutrient acquisition, and biofilm formation, thereby facilitating resource acquisition in complex environments for saprophytic or antagonistic fungi [41]. This differential distribution suggests that distinct biocontrol fungi exhibit species-specific characteristics in the construction of their proteolytic systems and in their functional emphases. In general, differences in peptidase family composition and abundance distribution among the biocontrol fungi reflect functional diversification in their modes of host interaction, nutrient-acquisition strategies, and ecological adaptation pathways. In addition to comparative genomic inference, we further analyzed the expression patterns of four highly secreted S8 serine protease genes (Alp1, PR1C, PR1D, and P32) under different carbon and nitrogen sources (Section 2.6, Figure 6). These results revealed differential transcriptional responses, providing direct experimental support at the transcriptional level for the proposed functional specialization and nutrient-responsive regulation of S8 proteases, which may facilitate host-associated interactions. While these data are preliminary and do not constitute full transcriptomic or proteomic validation, they strengthen the mechanistic framework inferred from genome analyses. In combination with the analyses of CAZymes and secondary metabolite gene clusters, it can be inferred that peptidases may act synergistically with polysaccharide-degrading enzymes and bioactive secondary metabolites, collectively suggesting a potential molecular basis for fungal participation in biocontrol processes. From a genomic perspective, this study provides new theoretical insights into the action strategies of different biocontrol fungi.

In addition to proteases, CAZymes represent another important functional module associated with host interaction. CAZymes, particularly glycoside hydrolases and chitinases, have been shown to contribute to the degradation of nematode eggshells and structural polysaccharides, thereby facilitating fungal colonization. In this study, the enrichment of GH and CBM families in *T. harzianum* is consistent with its well-documented capacity for plant and microbial cell wall degradation [40]. In contrast, the relative abundance of GT and PL families in *P. lilacinum* may facilitate surface modification and host interaction during nematode infection [32]. Together, these findings are consistent with the hypothesis that secondary metabolites and CAZymes may act synergistically during nematode antagonism. However, direct evidence from co-expression or gene knockout studies is needed to confirm this relationship [28,42].

Transporters and transcription factors provide a regulatory and functional framework supporting fungal biocontrol activity. Although no marked expansion of these components was observed, their conserved presence across all four fungi suggests a stable system for metabolite transport and gene regulation(Figure S3). In particular, Zn(2)-Cys(6) transcription factors are known to regulate secondary metabolism and enzyme expression, indicating their potential role in coordinating hydrolytic enzymes and metabolite production during host interaction [43,44].

Importantly, these functional components are unlikely to act independently; rather, they operate in a coordinated manner during nematode infection. From a mechanistic perspective, these functional modules may represent sequential and complementary steps during nematode infection. Proteases such as S8 serine peptidases may initiate host invasion by degrading proteinaceous barriers, followed by CAZymes that facilitate polysaccharide breakdown in eggshells and surrounding matrices. Subsequently, secondary metabolites may contribute to nematode mortality through toxic or inhibitory effects (Figure 8). This sequential and coordinated action provides a plausible mechanistic framework linking genomic features to biological function.

Despite these findings, this study is primarily based on comparative genomic analyses and lacks direct experimental validation. The functional roles of the identified gene families, including S8 proteases, CAZymes, and secondary metabolite gene clusters, remain to be confirmed through transcriptomic, proteomic, and gene knockout studies. Future work integrating multi-omics approaches will be essential to validate the proposed mechanisms.

In summary, this study provides an integrated comparative genomic framework linking hydrolytic enzymes and secondary metabolism in nematode biocontrol fungi. By identifying candidate functional modules and proposing a coordinated mechanistic model, our findings offer a foundation for future experimental studies aimed at improving the efficacy of fungal biocontrol agents.

## 4. Materials and Methods

### 4.1. Genome Data Collection

Genome sequences and corresponding annotation files of *P. lilacinum* PLFJ-1 [23], *T. harzianum* CBS 226.95 [25], *A. niger* CBS 513.88 [24], and *P. chlamydosporia* 170 [25] were obtained from publicly available databases, including the NCBI Genome database and the JGI MycoCosm platform. All genome datasets were processed under a unified framework to ensure comparability across species. Basic genome statistics, including genome size, GC content, scaffold number, and N50 values, were calculated based on the downloaded assemblies.

### 4.2. Orthologous Gene Family Analysis

Orthologous gene families were identified using OrthoFinder (v3.1.1) [45] with default parameters. Protein sequences from all four species were used as input. The resulting orthogroups were used to identify shared (core) and species-specific gene families. The UpSet [46] plot was generated using custom Python (3.1.0) scripts to visualize intersections among orthogroups.

### 4.3. Phylogenetic Analysis

To establish a broad phylogenetic framework for species selection in comparative genomic analyses, ITS sequences of 11 fungal species representing diverse ecological lifestyles, including nematophagous fungi, entomopathogenic fungi, biocontrol-associated fungi, and saprophytic *Aspergillus* species, were retrieved from public databases. Sequences were aligned using ClustalW [47] implemented in MEGA [48], and a Neighbor-Joining (NJ) tree was constructed with 1000 bootstrap replicates. Only bootstrap values >50% were displayed. This preliminary analysis supported the selection of four representative species (*P. lilacinum*, *P. chlamydosporia*, *T. harzianum*, and *A. niger*) for subsequent comparative genomic analyses.

Single-copy orthologous proteins shared among the four representative fungal genomes were identified using OrthoFinder, and 200 conserved orthologs were selected for concatenated phylogenomic analysis. Protein sequences were extracted, concatenated, and aligned. A maximum-likelihood phylogenetic tree was reconstructed using IQ-TREE [49] with 1000 bootstrap replicates to assess branch support. This high-resolution phylogenomic tree provides a robust evolutionary framework for subsequent comparative analyses of gene families associated with parasitism, host interaction, and biocontrol potential, including CAZymes, peptidases, and secondary metabolite-related genes.

For functional gene family analyses, including S8 serine proteases and chitinases, protein sequences were extracted from the four studied genomes. Multiple sequence alignment was performed using MUSCLE [50], and phylogenetic trees were reconstructed using IQ-TREE with 1000 bootstrap replicates. All phylogenetic trees were visualized using iTOL [51].

### 4.4. Functional Annotation

Protein sequences were functionally annotated against multiple databases: KEGG annotation was performed using KAAS (KEGG Automatic Annotation Server) [52], GO annotation was conducted using Blast2GO (v4.1) [53] and KOG classification was assigned using eggNOG-mapper (v2.1) [54]. Functional categories were summarized and visualized using R (ggplot2) [55].

Protein sequences from species-specific identified by OrthoFinder were functionally annotated using the eggNOG-mapper pipeline implemented in OmicsBox. Gene Ontology (GO) annotations were assigned to each predicted protein based on orthology inference and categorized into three functional domains: biological process (BP), molecular function (MF), and cellular component (CC).

GO enrichment analyses were conducted separately for species-specific genes. Enrichment significance was evaluated using a hypergeometric test by comparing the frequency of each GO term in the target gene set against its frequency in the whole-genome background. Enrichment significance was evaluated using Fisher’s exact test (hypergeometric distribution), and multiple testing correction was conducted using the Benjamini–Hochberg false discovery rate (FDR) method. Functional categories with adjusted *p*-values (FDR) < 0.05 were considered significantly enriched. Enriched GO categories were visualized as bar plots using Graphpad Prism (v10.1.2), with particular attention to functional groups potentially associated with parasitism, host interaction, nutrient utilization, and environmental adaptation.

### 4.5. Identification of CAZymes

Carbohydrate-active enzymes were identified using the dbCAN2 [56] meta server, which integrates HMMER, DIAMOND, and Hotpep methods. Only CAZyme families supported by at least two methods were retained to improve annotation accuracy. Identified CAZymes were classified into GH, GT, CE, PL, CBM, and AA families.

### 4.6. Identification of Peptidases

Peptidases were annotated using the MEROPS database (https://www.ebi.ac.uk/merops/). Protein sequences were searched against the MEROPS database using BLASTP (v2.17.0) with an E-value threshold of 1 × 10^−5^. Identified peptidases were classified into major categories, including serine, metallopeptidases, cysteine, and aspartic peptidases. S8 serine proteases were further extracted for phylogenetic analysis.

### 4.7. Secondary Metabolite Gene Cluster Prediction

Secondary metabolite biosynthetic gene clusters were predicted using antiSMASH (v6.0) [57] with default settings. Predicted clusters were classified into PKS, NRPS, PKS-like, NRPS-like, hybrid, and terpene clusters. The distribution of BGC types was summarized and visualized.

### 4.8. Analysis of Transporters and Transcription Factors

Transporter proteins were annotated using the Transporter Classification Database (TCDB) [58]. Transcription factors were identified based on conserved domains using InterProScan (v5) [59]. TF families, including Zn(2)-Cys(6), bZIP, C2H2, and helix-loop-helix domains, were classified according to domain composition.

### 4.9. Nutrient-Responsive Expression of S8 Serine Proteases

Spore suspensions of *P. lilacinum* were inoculated into PDB medium with different carbon sources (corn flour, soluble starch, sucrose, glucose) or nitrogen sources (skim milk powder, casein, peptone, yeast extract, ammonium sulfate) and incubated at 28 °C, 150 r/min. Samples were collected at 6, 12, 24, 36, and 48 h, centrifuged at 10,000 rpm for 10 min at 4 °C, and stored at −80 °C.

Total RNA was extracted and reverse-transcribed to cDNA using HiScript III RT SuperMix. qRT-PCR was performed for four highly secreted S8 serine protease genes (Alp1, PR1C, PR1D, P32) using ChamQ Blue Universal SYBR qPCR Master Mix (Vazyme Biotech Co., Ltd., Nanjing, China) on a 7500 Real-Time PCR System, with Actin as internal reference. Reactions were run in triplicate, and relative expression levels were calculated using standard curves. Data were visualized using GraphPad Prism (v 10.1.2).

## 5. Conclusions

In conclusion, this comparative genomic study reveals that biocontrol fungi share conserved core metabolic functions while exhibiting substantial divergence in gene families associated with host interaction and nematode antagonism. In particular, the differential enrichment of S8 serine peptidases, CAZyme repertoires, and secondary metabolite biosynthetic gene clusters suggests that these fungi may employ distinct yet coordinated molecular strategies for host recognition, penetration, and degradation. These findings support a hypothetical multi-layered biocontrol mechanism integrating hydrolytic enzyme activity and metabolite-mediated antagonism. Importantly, this study provides an integrated genomic framework and identifies candidate functional modules for future transcriptomic and experimental validation. Since this study was based primarily on comparative genomic prediction, transcriptomic, proteomic, and functional validation analyses are still required to confirm the biological roles of the identified genes and pathways in fungal biocontrol mechanisms.

## Figures and Tables

**Table 1:** Genome assembly and annotation statistics. “–” indicates data not available or not reported in the original reference.

Genome Characteristics	*Aspergillus niger* CBS 513.88	*Purpureocillium lilacinum* PLFJ-1	*Trichoderma harzianum* CBS 226.95	*Pochonia chlamydosporia* 170
Genome size	34 Mb	38.5 Mb	41 Mb	44.2 Mb
Total ungapped length	33.9 Mb	38.2 Mb	41 Mb	44.2 Mb
Number of scaffolds	19	163	532	49
Scaffold N50	2.5 Mb	3.2 Mb	2.4 Mb	5.4 Mb
Scaffold L50	6	5	7	4
Number of contigs	469	680	841	114
Contig N50	114 kb	150.1 kb	360.6 kb	2 Mb
Contig L50	96	76	38	8
GC percent	50.5	58.5	47.5	49.5
Genome coverage	7.5×	152×	120×	211.0×
Assembly level	Scaffold	Scaffold	Scaffold	Chromosome
Number of chromosomes	8	–	–	7
Genome accession number	AM270980-AM270998	LSBI00000000	Not reported	Not reported
Reference	[23]	[24]	[25]	[25]

## Data Availability

The original contributions presented in this study are included in the article. Further inquiries can be directed to the corresponding authors.

## Supplementary Materials

The following supporting information can be downloaded at https://www.mdpi.com/article/10.3390/ijms27114687/s1.
