# Supplementary figures and images for: Comparative Genomics Analysis Reveals the Genomic Basis of S8 Proteases, CAZymes, and Secondary Metabolism Associated with Nematode Biocontrol in Purpureocillium lilacinum
Source: Int J Mol Sci. 2026 May 22;27(11):4687. doi: 10.3390/ijms27114687 (PMC13256968; doi:10.3390/ijms27114687)

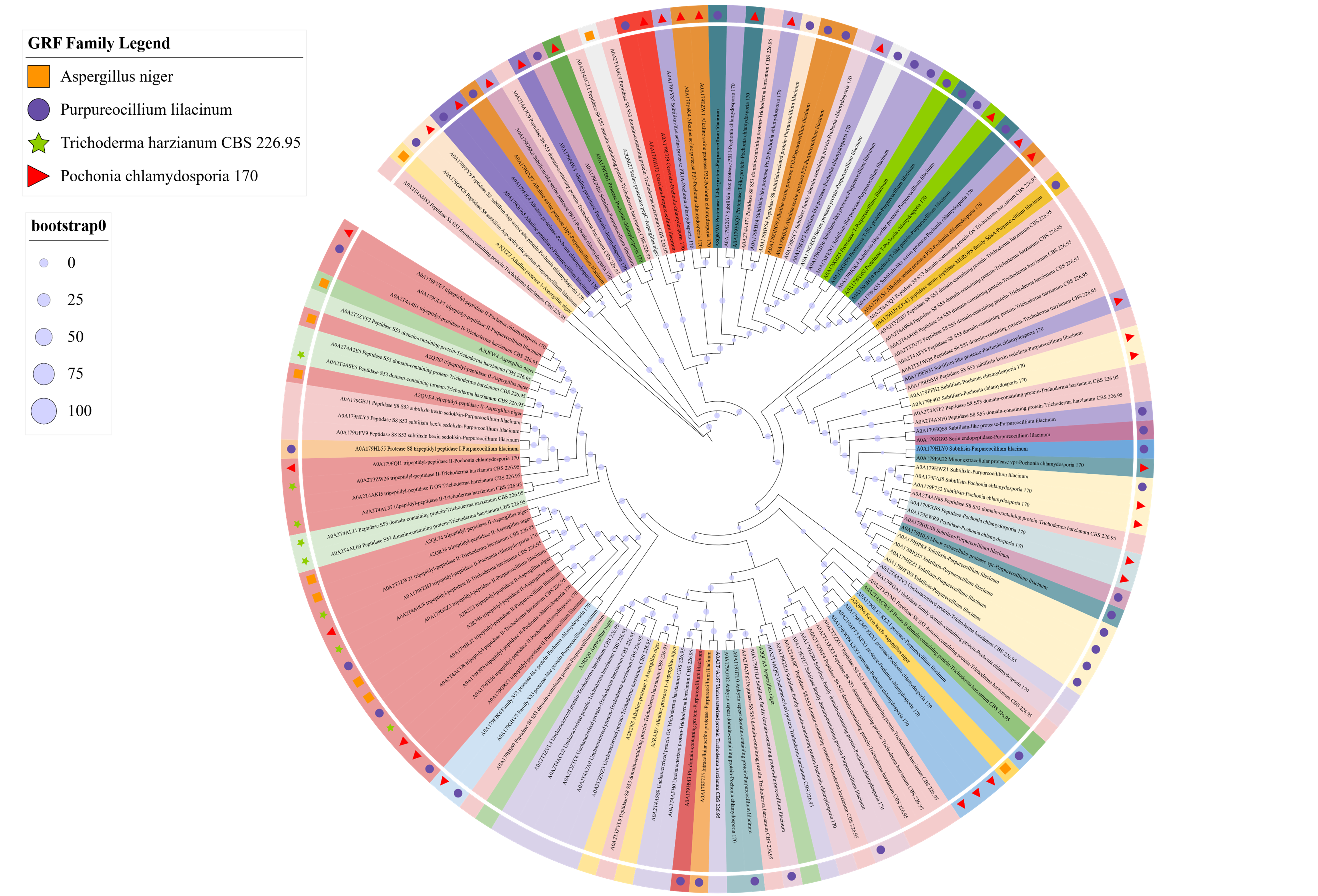

Supplement: Supplementary file 1 [file ijms-27-04687-s001.zip › Supplementary/Figure S1.tif]

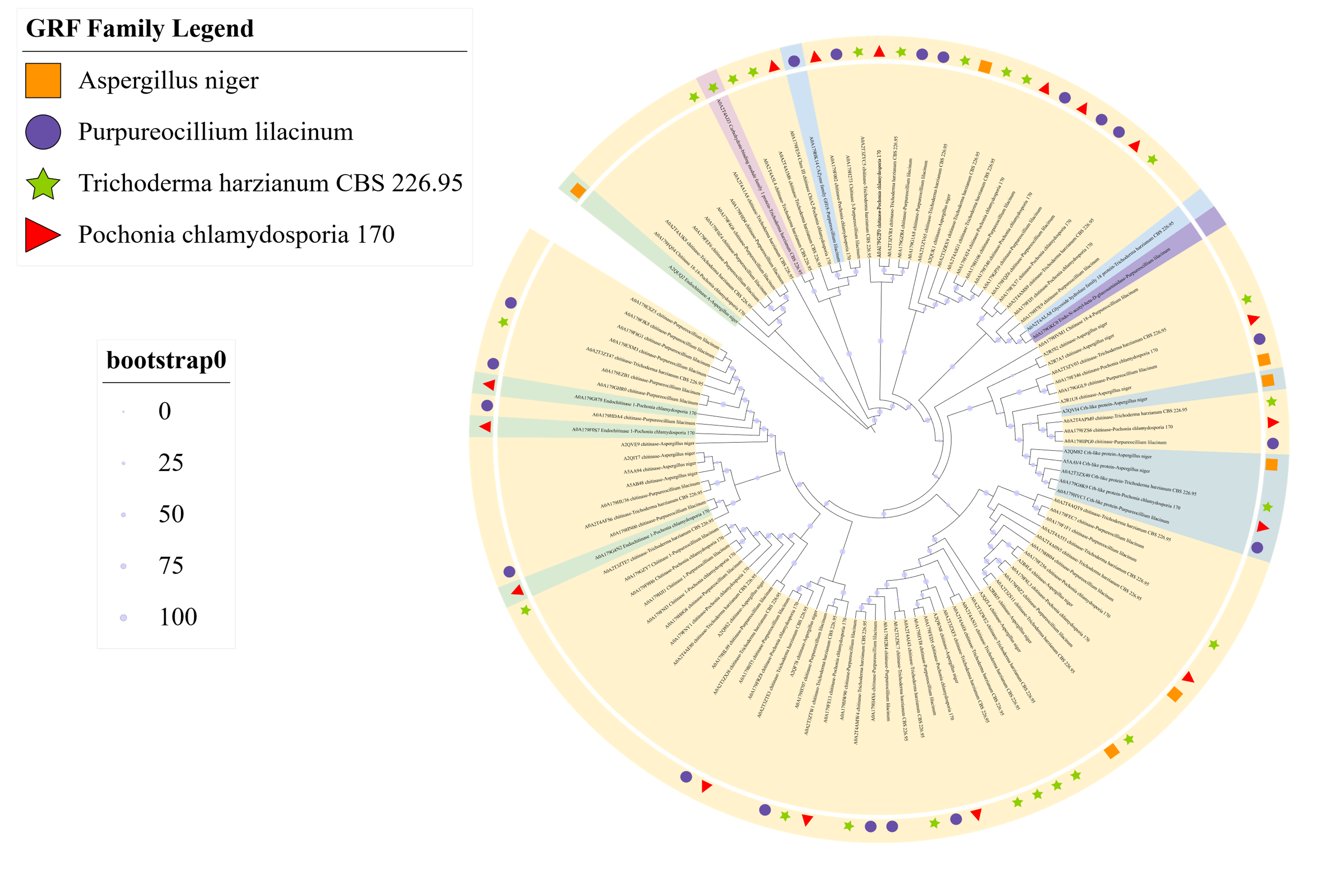

Supplement: Supplementary file 1 [file ijms-27-04687-s001.zip › Supplementary/Figure S2.tif]

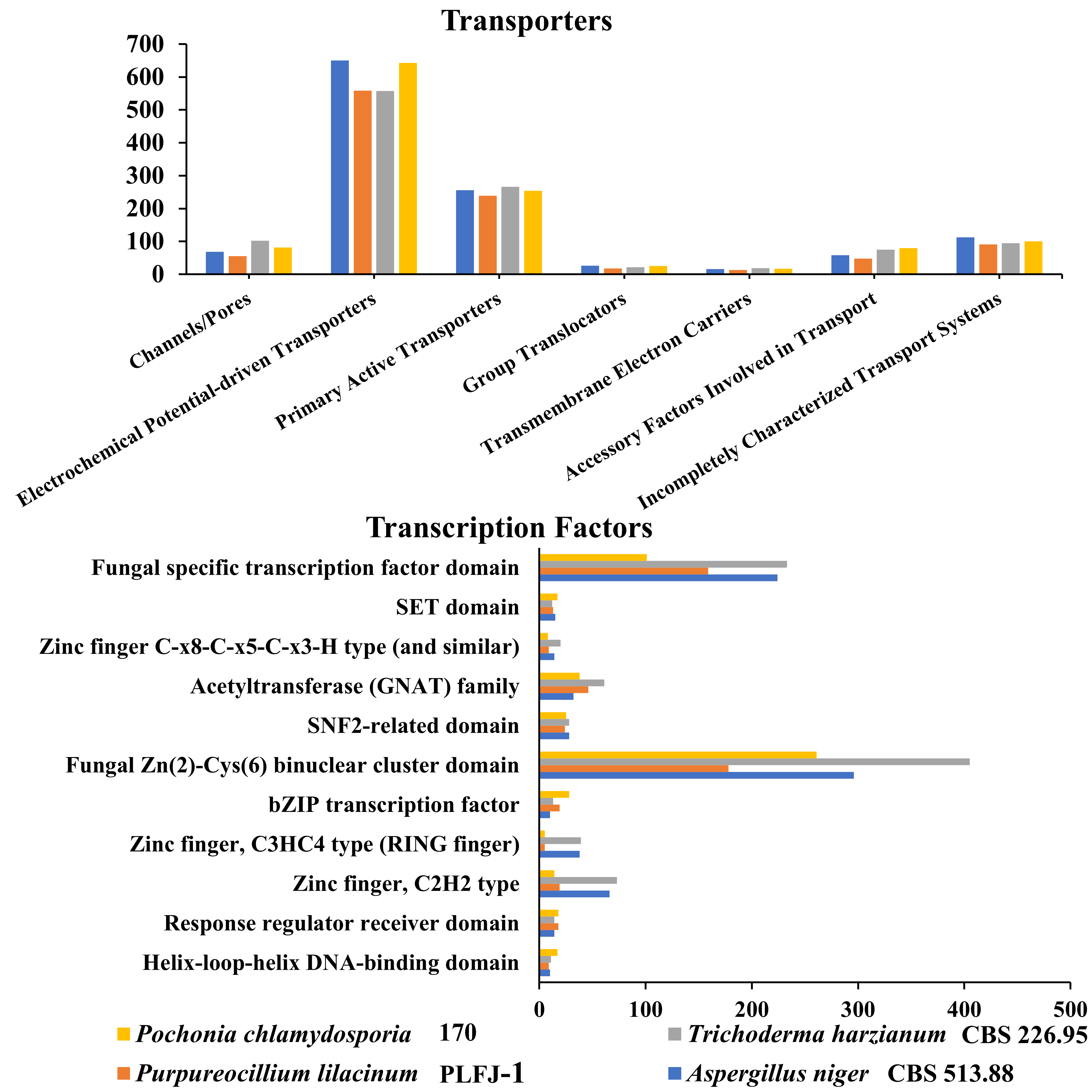

Supplement: Supplementary file 1 [file ijms-27-04687-s001.zip › Supplementary/Transporters and Transcription Factors.tif]
